# Characterization of novel recombinant mycobacteriophages derived from homologous recombination between two temperate phages

**DOI:** 10.1093/g3journal/jkad210

**Published:** 2023-09-15

**Authors:** Hamidu T Mohammed, Catherine Mageeney, Jamie Korenberg, Lee Graham, Vassie C Ware

**Affiliations:** Department of Biological Sciences, Lehigh University, Bethlehem, PA 18015, USA; Memsel, Inc., 3500 Camp Bowie Blvd., Fort Worth, TX 76107, USA; Department of Biological Sciences, Lehigh University, Bethlehem, PA 18015, USA; Biotechnology and Bioengineering Department, Sandia National Laboratories, Livermore, CA 94551, USA; Department of Biological Sciences, Lehigh University, Bethlehem, PA 18015, USA; New York Institute of Technology College of Osteopathic Medicine, 101 Northern Blvd., Glen Head, NY 11545, USA; Department of Biological Sciences, Lehigh University, Bethlehem, PA 18015, USA; Department of Biological Sciences, Lehigh University, Bethlehem, PA 18015, USA

**Keywords:** mycobacteriophage genomics, phage genome mosaicism, homologous recombination, phage genetic diversity, bacteriophage evolution, phage therapy

## Abstract

Comparative analyses of mycobacteriophage genomes reveals extensive genetic diversity in genome organization and gene content, contributing to widespread mosaicism. We previously reported that the prophage of mycobacteriophage Butters (cluster N) provides defense against infection by Island3 (subcluster I1). To explore the anti-Island3 defense mechanism, we attempted to isolate Island3 defense escape mutants on a Butters lysogen, but only uncovered phages with recombinant genomes comprised of regions of Butters and Island3 arranged from left arm to right arm as Butters-Island3-Butters (BIBs). Recombination occurs within two distinct homologous regions that encompass *lysin A, lysin B*, and *holin* genes in one segment, and *RecE* and *RecT* genes in the other. Structural genes of mosaic BIB genomes are contributed by Butters while the immunity cassette is derived from Island3. Consequently, BIBs are morphologically identical to Butters (as shown by transmission electron microscopy) but are homoimmune with Island3. Recombinant phages overcome antiphage defense and silencing of the lytic cycle. We leverage this observation to propose a stratagem to generate novel phages for potential therapeutic use.

## Graphical Abstract

**Graphical Abstract:**
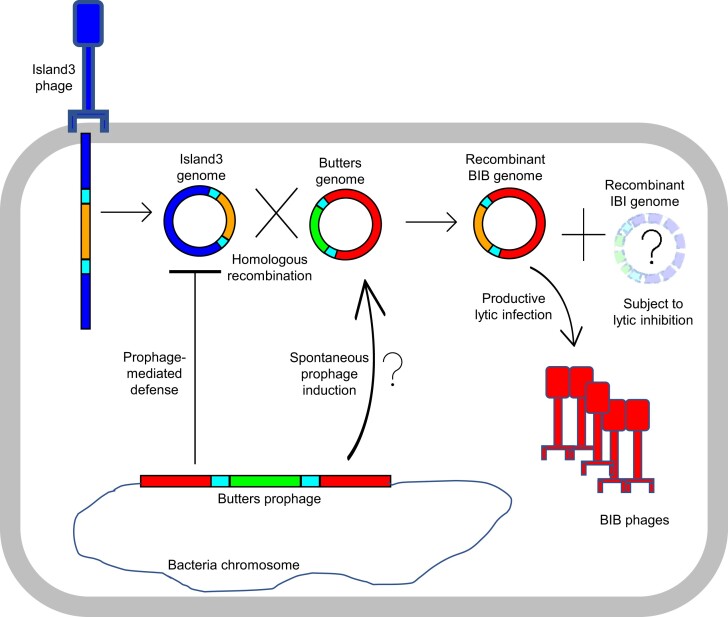

## Teaser

*In silico* comparative analysis of mycobacteriophage genomes reveals extensive genetic diversity and widespread genome mosaicism. Mohammed et al., describe a novel mosaic recombinant phage named BIB (Butters-Island3-Butters) produced *in situ* by homologous recombination between the Mycobacteriophage Butters (cluster N) prophage within a lysogen and the invading Island3 (subcluster I1) genome. Mycobacteriophage Butters prophage-encoded factors block lytic infection of heterotypic Mycobacteriophage Island3 but the recombinant BIB genome contains genetic modules that allow escape from prophage-mediated defense and repressor-mediated lytic repression.

## Introduction

The uptick in phage biology research coupled with a more accessible sequencing regime has led to sequencing thousands of phage genomes (Reyes et al. 2012). Comparative genome analysis reveals extensive mosaicism that characterizes phage genomes (Mavrich and Hatfull 2017; Oliveira et al. 2019; Yahara et al. 2019). Phage genomes are conceived of as a patchwork of exchangeable genetic modules that are shuffled between phages via horizontal gene transfer (Botstein 1980). Phage genome mosaicism is generated mechanistically by homologous recombination (Clark et al. 2001; Martinsohn et al. 2008; De Paepe et al. 2014) or by illegitimate recombination (Hendrix et al. 2000; Juhala et al. 2000; Pedulla et al. 2003; Morris et al. 2008). With either recombination mechanism, the opportunity for recombination between phage genomes would be presented in a host cell during coinfection or when an invading phage infects a lysogen (Hendrix et al. 2000).

The prevalence of prophages in bacterial genomes (Casjens 2003; Touchon et al. 2016) likely increases the probability of an invading phage genome co-occurring along with a resident prophage, creating an avenue for recombination between two phage genomes. Some prophages collude with their host to defend against heterotypic phage infection (Labrie et al. 2010; Bondy-Denomy et al. 2016; Dedrick et al. 2017; Hampton et al. 2020). It is theorized that genetic recombination between phages may be an evolutionary strategy for phages to exchange antiphage defense systems (Piel et al. 2021; Kauffman et al. 2022).

The availability of large collections of sequenced mycobacteriophages provides opportunity to observe and investigate the relationship between phage genome mosaicism and exchange of antiphage defense systems. The HHMI-sponsored SEA-PHAGES program has led to the isolation of ca. 12,579 mycobacteriophages with over 2,000 genomes fully sequenced (“The Actinobacteriophage Database | Home”; Russell and Hatfull 2017; Hatfull 2020). These genomes are extensively mosaic (Pedulla et al. 2003; Pope et al. 2015). Mycobacteriophages are currently grouped into 31 clusters (A–Z, AA–AE) based on genome similarity and singletons with no known close relatives (“The Actinobacteriophage Database | Home”; Cresawn et al. 2011; Pope et al. 2015; Russell and Hatfull 2017). Although genomes are categorized into clusters, many genes and groups of genes are shared between clusters, thereby contributing to extensive genome mosaicism.

Cluster N mycobacteriophages, in particular, have been highlighted for the diversity that characterizes the central region of their genomes between more conserved sequences in the left and right arms (Dedrick et al. 2017). Genes within the central region of cluster N mycobacteriophages are expressed in cluster N lysogens and have been implicated in prophage-mediated defense mechanisms that defend the host from heterotypic viral attack (Dedrick et al. 2017; Mageeney et al. 2020). Cluster N and I1 mycobacteriophage genomes are notable in their continuum of genetic similarity and are proposed to exchange genetic modules at a relatively faster rate (Mavrich and Hatfull 2017). Despite extensive in silico characterization of mosaicism within mycobacteriophage genomes (Pedulla et al. 2003; Morris et al. 2008; Dedrick et al. 2017; Mavrich and Hatfull 2017), relatively little direct experimental evidence exists to document how mosaicism emerges. Thus, gaps remain in our understanding of mechanisms underlying development of genome mosaicism and in the biological consequences of this phenomenon among mycobacteriophages.

Here, we describe the isolation and characterization of recombinant mycobacteriophages formed between a Butters (cluster N) prophage and an infecting Island3 (subcluster I1) genome. These mosaic phages have been named BIBs to reflect the source of genetic segments that constitute their genome from left to right (Butters-Island3-Butters). Our data reveal a biological phenomenon where homologous recombination provides a means for the invading phage to overcome antiphage defense and the resident phage to escape from lytic repression. We discuss this phenomenon in the context of phage therapy as a measure where mixed phage lysates in coinfections are used to control bacterial infections.

## Materials and methods

### Phage isolation, propagation, and genomic analysis

Phages (GenBank accession numbers KC576783 [Butters; Sin et al. 2013], HM152765 [Island3; Alcoser et al. 2010], and MN945904 [Eponine; Mansi et al. 2020]) were isolated and grown on *Mycobacterium smegmatis* mc^2^155 as described in Caratenuto et al. (2019). Island3 lysate was obtained from the Hatfull lab (University of Pittsburgh). The genomic sequence for the Island3 strain used in this study differs from that of the wild type with a 257-bp deletion (coordinates 43,307 to 43,563) and a C2,656 T SNP. DNA for recombinant BIBs were isolated using phenol:chloroform extraction. BIBs 1–4 were sequenced at the Pittsburgh Bacteriophage Institute, Pittsburgh, PA, and BIBs 6–10 were sequenced at Novogene Corporation Inc (Russell 2020). Phage lysates (titers, **≥** 1 × 10^9^ PFU/ml), diluted with phage buffer (0.01 M Tris [Mohammed and Ware 2022], pH 7.5, 0.01 M MgSO_4_, 0.068 M NaCl, and 1 mM CaCl_2_), were used for immunity testing and PCR. Phamerator (Cresawn et al. 2011) was used for comparative genomic analysis and genome map representation. DefenseFinder (https://defense-finder.mdmparis-lab.com/) was run on the webserver using the nucleic acid with CRISPR array detection (Abby et al. 2014; Tesson et al. 2022). PADLOC (https://padloc.otago.ac.nz/padloc/myjobs/) was run at the website using the nucleotide fasta option (Payne et al. 2022).

### Phage sensitivity assays

*Spot test assay for immunity tests:* Lawns of *M. smegmatis or* lysogens were made by plating 250 µl of the bacteria culture with 3.5 ml of top agar (at ∼55°C) on an LB agar plate (with 10 µg/ml of cycloheximide [CHX] and 50 µg/ml of carbenicillin [CB]). Phage lysates were serially diluted to 10^7^ and spotted (3 µl each) onto lawns of interest. Plates were incubated for 48 h at 37°C. Phage growth was assessed at 24 and 48 h. *Plaque assays:* Phage lysates were serially diluted. Ten microliters of each dilution was added to 250 µl of the culture or lysogen to be infected and incubated for 10 minutes at room temperature. Top agar (3.5 ml) was added to the phage-bacteria mix and plated on LB agar plates (CHX/CB).

### Electron microscopy

Phage lysate was deposited onto 200 mesh copper grids coated with formvar and carbon (Ted Pella: 01810), rinsed with deionized water and stained with 2% ammonium molybdate. Samples were imaged on a JEOL 1200EX transmission electron microscope (TEM). Capsid and tail length measurements were done using ImageJ (Schneider et al. 2012). Measurements represent the average of at least 6 particles.

### Polymerase chain reactions

Samples were derived from pure phage lysates or plaques picked into 100 µl of phage buffer. Platinum™ II Hot-Start Green PCR Master Mix (2X) (Invitrogen) was used. One microliter of each sample was used as template. Primers used are listed in Supplementary Table 1. All primers were ordered from Integrated DNA Technologies, Inc.

### Determination of number of secondary plaques to screen

Assuming 1 out of 10 phages within a plaque is non-BIB (i.e. either Butters, Island3, or the reciprocal product: Island3-Butters-Island3 [IBI]), then the probability of obtaining 1 non-BIB phage at 95% confidence interval is given by:

$1-{\left(\frac{9}{10}\right)}^{n}\;=\;0.95$ , where *n* is the sample size of plaques to be screened to achieve a 95% confidence interval. From this equation we calculated that *n* must be at least 28.5 (rounded up to 30).

## Results

Mycobacteriophage Butters is a temperate phage that encodes antiphage defense systems against several heterotypic phages (Dedrick et al. 2017). Analysis of Butters genome using DefenseFinder (Abby et al. 2014; Tesson et al. 2022) and PADLOC (Payne et al. 2022) yielded no hits, suggesting that Butters-encoded defense systems are likely novel. RNAseq profile of Butters lysogen showed that Butters prophage genes *30–38*, and the antisense strands of genes *42–43* and gene *57* are expressed (Dedrick et al. 2017). Analyses of the roles of these genes in defense showed that Butters gp30 and gp31 form a two-component system that inhibits infection by mycobacteriophage PurpleHaze but not Island3 (Mageeney et al. 2020). Further, the antisense strand of Butters gene 57 (gp57r) was found to mediate defense against Island3 and a host of other phages (Mohammed et al. 2023). While the mechanism of gp57r defense is not known, it is predicted to act downstream of the DNA injection stage and its antagonism leads to a reduction in the quantity of Island3 genomes made during infection (Mohammed et al. 2023). To explore the mechanism of defense and to identify putative Island3 targets of Butters prophage defense, we pursued isolation of Island3 defense escape mutants (DEMs)—mutants that have escaped Butters prophage-mediated defense and consequently, can efficiently infect a Butters lysogen [mc^2^155(Butters)].

**Fig. 1.**
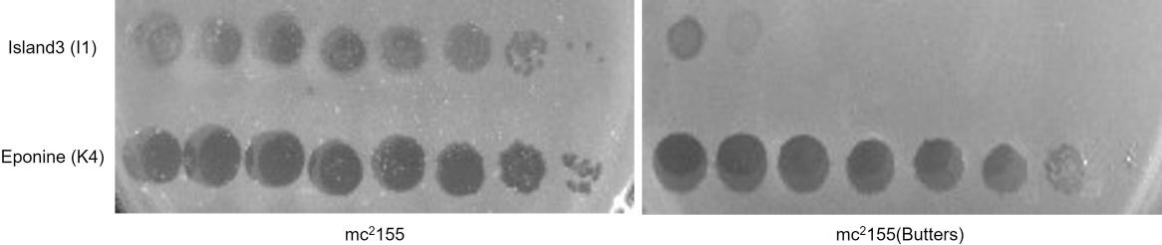
Phage sensitivity assay (spot tests) showing mc^2^155 (butters) defense against Island3. Ten-fold serial dilutions (3 µl) of Island3 and Eponine (as control) were spotted onto lawns of *Mycobacterium smegmatis* mc^2^155 (mc^2^155) and Butters lysogen [mc^2^155(Butters)].

Single Island3 plaques, which typically constitute evidence of mutants, were not observed when serial dilutions of an Island3 high titer lysate were spotted on a Butters lysogen lawn (Fig. 1). Prominent spot clearings of high Island3 concentrations are regarded as “killing from without” rather than evidence of successful infections (Delbrück 1940; Abedon 2011). Yet, it is conceivable that clearings associated with “killing from without” may mask individual plaques arising from an Island3 DEM. To explore this possibility, plaque assays were performed. Typically, about 10 plaques are formed upon infecting Butters lysogen with 10^8^ pfu of Island3. Nine of these putative Island3 DEMs were successfully sequenced.

### Recombinant plaques arise from a double homologous recombination event between Butters and Island3 genomes

**Fig. 2.**
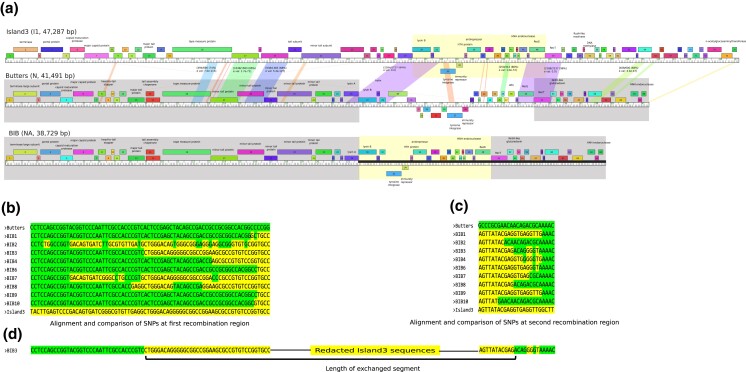
Genome characterization of Island3, butters, and recombinant phage, BIB. a) Phamerator maps (Cresawn et al. 2011) for Butters, and Island3 and a derived map for BIB. Genes are depicted by rectangular boxes and are aligned to their genomic ruler. Gene numbers are within the rectangular boxes and putative gene functions, if known, are indicated above the gene. Sections defined between genomes show nucleotide similarity between the genome segments with variable E value scores. Selected segments with over 300 bp aligned sequences and E value ≤ 1e−20 are shown within connecting sections. Within each square bracket is the fraction of number of nucleotide identities over total aligned sequences, percentage of nucleotide identity in parentheses and E value (e val). The bottom row is a representative map of a BIB genome. Blocks or modules derived from Butters are shaded darker and the module derived from Island3 is shaded lighter. b) Alignment of SNPs across the first homology region. The entire nucleotide sequences that defines the first homology region (genome coordinates: 21,825–23,917 [Butters] and 25,302–27,409 [Island3]) were aligned in Jalview (Waterhouse et al. 2009). Conserved sequences were redacted to yield only SNPs. From left to right, sequences of BIBs first map to Butters before transitioning into Island3. SNPs in some BIBs “flipflop” from one parental genome to another and back. c) Alignment of SNPs across the second homology region (genome coordinates: 33,050–35,516 [Butters] and 33,774–35,854 [Island3]. From left to right, BIB genomes align to Island3 before transitioning into Butters. d) Lengths of genetic segments exchanged during recombination. Length of Island3 module was estimated from the first occurrence of SNP originating from the Island3 genome in the first homology region to the first occurrence of a SNP originating from the Butters genome in the second homology region. The same coordinates were used to estimate the length of the Butters module that was swapped out from the Butters genome.

Sequencing of putative Island3 DEMs showed that genomes are recombinants with the left arm contributed by Butters, the middle segment derived from Island3, and the right arm contributed by Butters (Fig. 2a). These recombinant phages are termed Butters-Island3-Butters (BIBs).

Comparative genome analysis reveals that BIBs are a product of a double homologous recombination event within two homologous regions between Butters and Island3. The first homologous region has an average nucleotide identity (ANI) of about 84% and spans about 2,727 bp of both genomes (Fig. 2a). This homology region contains 4 predicted open reading frames (ORFs) or protein coding genes; lysin A, lysin B, holin, and a gene of unknown function (Butters gene *29* or Island3 gene *32*) (Fig. 2a). By mapping single nucleotide polymorphisms (SNPs) originating from Butters vs those originating from Island3 to BIB genome sequences, we established that the switch from the Butters genome to Island3 genome occurs within lysin B (Fig. 2b). The second homology region has an ANI of about 98% and spans about 2,237 bp of both genomes (Fig. 2a). This second homology region contains three ORFs: a gene of unknown function (Butters gene *49* or Island3 gene *47*), RecE, and RecT (Fig. 2a). Here, the switch from one genome to the other is variously distributed within RecE and RecT (Fig. 2a). We estimated the length of Island3 segment incorporated in the mosaic genome by determining the nucleotide distance from the first instance of a SNP in a BIB that originates from the Island3 genome in the first homology region to the first instance of a SNP that originates from the Butters genome in the second homology region. Lengths of incorporated Island3 segments ranged from 6,722 bp to 8,854 bp (Table 1), while the corresponding length of Butters replaced in the mosaic BIB genomes was estimated to range from 9,484 bp to 11,616 bp (Table 1). In all cases, the resulting BIB genome is 2,762 bp shorter than the Butters genome (Fig. 2a, Table 1).

**Table 1.** Lengths of genetic segments exchanged in each BIB.

Recombinant BIB	Island3 module size (bp)	Butters module size (bp)	Difference
BIB1	8,797	11,559	2,762
BIB2	7,543	10,305	2,762
BIB3	7,483	10,245	2,762
BIB4	7,826	10,588	2,762
BIB6	8,070	10,832	2,762
BIB7	8,649	11,411	2,762
BIB8	8,448	11,210	2,762
BIB9	8,854	11,616	2,762
BIB10	6,722	9,484	2,762

### Recombinant BIB phenotypic characteristics are determined by specific parental phage genomic modules

**Fig. 3.**
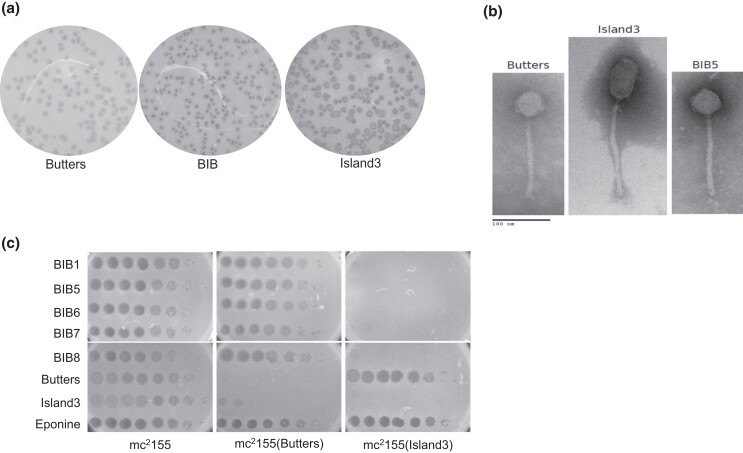
Physical and immunity properties of BIBs and their parental phages. a) Comparison of plaque morphologies of Butters, Island3, and BIBs. Plaque assay was performed by plating serial dilutions of the phage lysates onto mc^2^155 and plaque morphologies assessed. BIBs present an emergent phenotype distinguishable from that of both Butters and Island3. b) Electron micrograph of Butters, Island3 and BIB phages. BIBs and Island3 share identical capsid and tail lengths. c) Immunity relationships for Butters, Island3, and BIBs. Three microliters of serially diluted phages were spotted on lawns of mc^2^155, mc^2^155(Butters), and mc^2^155(Island3). BIBs are homoimmune with Island3.

Morphological and immunity characteristics of recombinant BIBs were compared to their parental phages. Plaque morphologies of mycobacteriophages differ on different media (Ramesh et al. 2019); plaque morphologies described here were determined on 7H10 agar. After ∼36–48 hours of incubation, Butters forms 2.0–3.0 mm turbid plaques with a translucent center encircled by a cloudier ring (Fig. 3a). Island3, on the other hand, forms 2.0–2.5 mm diameter bullseye plaques with three distinct regions: a clear center, a cloudy ring enveloping the clear center, and a clear outer ring (Fig. 3a). Interestingly, BIBs show an emergent plaque morphology, distinguishable from those of Butters or Island3. While BIBs form bullseye plaque morphologies like those of Island3, the clear centers of BIB plaques are relatively larger while the middle cloudy rings are significantly smaller (Fig. 3a). It therefore appears that genes within the exchanged module from Island3 play a significant part in determining plaque morphology phenotype; however, contributions from the non-Island3 part of the BIB genome cannot be ruled out.

Given that BIBs derive all structural assembly genes from Butters and the immunity cassette from Island3, we hypothesized that BIBs will be morphologically identical to Butters and homoimmune with Island3. TEM images show both BIBs and Butters have an icosahedral head with a diameter of 53.0 ± 4.0 nm while Island3 has a prolate head with longitudinal height of 82.0 ± 2.0 nm and latitudinal width of 41.0 ± 2.0 nm (Fig. 3b). Further, both BIBs and Butters have tail lengths of 169.0 ± 5.0 nm while that of Island3 is 202.0 ± 2.0 nm (Fig. 3b). These results are not surprising, as both major capsid protein (which defines capsid diameter) and tape measure protein (which defines tail lengths) of BIBs are contributed by Butters. Immunity experiments performed by spotting dilutions of BIB lysates on lawns of *M. smegmatis* mc^2^155, mc^2^155(Butters), and mc^2^155(Island3) show that BIBs efficiently infect mc^2^155(Butters) but are inhibited on an mc^2^155(Island3) lawn, due to Island3 immunity repressor activity (Fig. 3c).

### BIB plaques are the predominant type from Island3 infection of mc^2^155(Butters)

**Fig. 4.**
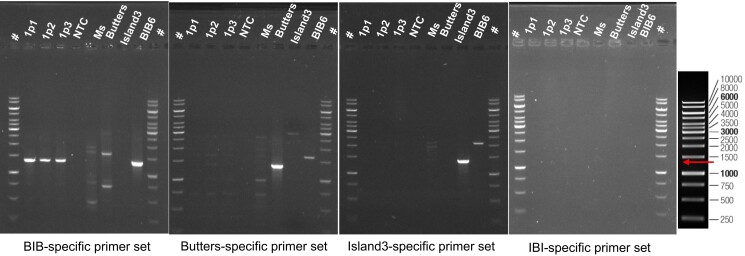
PCR screening of plaques obtained from plating experiments. Primers specific for each possible phage type (BIB, Butters, Island3, and IBI) for first homology region were designed and used to screen plaques via PCR. PCR products were run on 0.8% agarose gel at 60 V. GeneRuler 1 kb DNA ladder, ThermoFisher Scientific (extreme right panel) was used as ladder (#). Expected product size is 1,367 bp for BIB (arrow). Nonspecific bands appear in some instances due to nonspecific primer annealing for PCR conditions used. Also, some of the nonspecific bands can be mapped to the mc^2^155 genome which is inevitably present when crude phage lysates are filtered through 0.2 µm filters. Samples from left to right are ladder, primary plaques (1p1, 1p2, and 1p3), no template control, *M. sm*e*gmatis* mc^2^155 (*Ms*), Butters lysate, Island3 lysate, BIB6 lysate, and ladder (#). Note that primary plaques show PCR products with BIB-specific primers but not with Butters- or Island3- or IBI-specific primers. This is representative of at least 3 independent biological replicates.

Since 100% of plaques isolated and sequenced were recombinant BIBs, we investigated whether plaques produced from Island3 infection of mc^2^155(Butters) were of mixed types that included wild type forms of Island3 and Butters and the reciprocal recombinant product IBI. Plaque assays were performed using Island3 to infect mc^2^155(Butters) to generate primary plaques. Primers were designed that would specifically PCR amplify each phage type across the recombinant regions (Supplementary Table 1). All primary plaques showed a BIB-specific product but were negative for the other predicted phage types (Fig. 4a, data shown for PCR screening across first homology region). Additionally, genome sequencing confirmed exchange between both homology regions (see BIB GenBank accession numbers provided in Data Availability). To test the possibility that the experimental set up may fail to detect other phage types present at low concentration, a null hypothesis was established, predicting that there would be a ratio of at least one phage type of Island3, IBI, or Butters to nine BIBs within a primary plaque. If the hypothesis is supported, screening 30 secondary plaques resulting from plating a primary plaque on mc^2^155 should yield at least 1 Island3, IBI, or Butters plaque at a 95% confidence interval (see methods). Plaque assays for 3 primary plaques were performed to generate secondary plaques on mc^2^155. In all cases, all thirty plaques were positive for a BIB-specific band but negative for all other phage types [Supplementary Fig. 1 (data for one set shown)]. The lack of other phage types essentially rules out the possibility of amplification of the Island3 genome prior to recombination as well as any amplification of an IBI genome, if formed.

## Discussion

The phenomenon of extensive mosaicism is well documented within phage genomes in general and within mycobacteriophages in particular (Mavrich and Hatfull 2017; Oliveira et al. 2019; Yahara et al. 2019). Evidence supporting mosaicism within mycobacteriophage genomes is derived largely from bioinformatic analyses of published genomes. While informative, these post facto analyses reveal little, if any, information about factors and evolutionary pressures that trigger or limit the occurrence of recombination between phage genomes that lead to mosaicism. Here, wet-lab evidence shows homologous recombination between two temperate mycobacteriophages, thus providing the springboard for a more mechanistic interrogation of how genomic mosaicism is generated within mycobacteriophages.

Comparative genome analysis suggests that the observed was produced from homologous recombination events at two homologous regions between the Butters prophage and Island3 genomes. Over the entire genome, Butters and Island3 have 68% ANI. While there are several other homologous regions across the two genomes with ANI ranging from 43 to 80%, all sequenced BIBs recombined at the two regions with the highest ANI of 84 and 98%, respectively. It is unknown if recombinant phage form by homologous recombination at a single homologous region (to yield BI or IB) or by recombination at any other potential homologous regions, as no evidence of these possibilities was apparent.

Structural assembly genes determine phage morphotype (Katsura and Hendrix 1984). Expectedly, EM morphology of BIBs is similar to that of Butters with a comparable tail length and head diameter (Fig. 3b). Island3, on the other hand presented a longer tail length and a prolate head (Fig. 3b). Homoimmunity of BIBs with Island3 was also expected since the BIB immunity cassette is derived from Island3. Several factors affect plaque morphology including lysogeny (Levine 1957; Shin et al. 2014), the presence of depolymerases on tail proteins (Pires et al. 2016), phage tolerance (Tzipilevich et al. 2021), and variation of media or growth conditions (Ramesh et al. 2019). All experiments were carried out on 7H10 plates using the same batch of mc^2^155 for each biological replicate. BIBs plaques are morphologically similar to those of Island3, yielding bullseye plaques in both instances, but the defining concentric circles are markedly different in areas. Clearly, the exchanged genetic module derived from Island3 contributes genes that significantly affect plaque morphology phenotype. Yet, the inability to fully recapitulate the Island3 plaque morphology in BIBs suggests that other genes native to Butters portions of BIBs play a role in determining plaque morphology.

Homologous recombination events are generally regarded as chance events which will occur given presence of homology regions and recombineering enzymes. Biological phenomena that drive recombination between phage genomes are poorly understood, but a recent study reported that the *Bacillus subtilis* prophage SPβ recombines with phi3T DNA during sporulation, but not in the absence of sporulation (Dragoš et al. 2021). It has been suggested that recombination is a means for phages to escape antiphage defenses (Piel et al. 2021; Kauffman et al. 2022). In support of this, Piel et al. (2021) showed that Vibrio phages escape antiphage defenses by exchanging antidefense genes through homologous recombination. While we do not know of any factors that promote the observed recombination phenomenon in this study, the outcome of recombination is that BIB phages escape both Butters prophage-mediated defense that targets Island3 and Butters repressor-mediated defense that inhibits Butters lytic cycle.

Recombinant IBIs were not recovered from infections of Island3 on a Butters lysogen. This is not surprising as we would expect an IBI to be inhibited from the repressive action of Butters immunity repressor within our experimental setup. Moreover, the Island3 portions of an IBI may harbor the Island3 factor(s) targeted by the Butters prophage-mediated defense system which will constrain its replication (Mohammed et al. 2023). Also, within the exchanged modules, Island3 genes *33 (integrase), 34 (repressor), 35 (HTH protein), and 36 (antirepressor)* are annotated to function in lysis-lysogeny regulation. These genes are replaced by Butters genes *37 (integrase), 38 (repressor), and 39 (excise)* which modulate lysis-lysogeny outcomes in Cluster N mycobacteriophages (Broussard et al. 2013). Island3 genes *37, 41, 47, and 48 (RecE)* have homologs within the Butters exchange module corresponding to Butters genes *40, 45, 49, and 50 (RecE),* respectively. Crucially, Island3 genes *38–40*, *42–45, 46 (*a type of *HNH endonuclease)* have no known homologs within the Butters module. If any of these Island3 genes play essential roles within the lytic cycle of Island3, then absence of a functional Butters homolog within the Butters module would also account for IBI nonviability. Finally, while recombination may be pervasive among groups of phages and lead to an increase in gene content variation, genome size remains conserved (Piel et al. 2021), presumably due to constraints imposed by capsid size. The Butters exchange module is 2,726 bp larger than the Island3 exchange module. This size increase would augment the genome of a hypothetical IBI to 50,049 bp instead of 47,287 bp. It is unknown if the prolate head of a recombinant IBI phage could accommodate the increase in genome size.

This work parallels that of De Paepe et al. (2014) where recombination between invading temperate phages and cryptic prophages in *E. coli* was observed. Their findings supported the proposition that recombinants arise after several rounds of phage genome amplification (Visconti and Delbrück 1953). Failure to detect Island3-specific PCR products within primary plaques is due to the presence of a prophage-mediated system which inhibits the generation of infective Island3 particles. The interplay between the Butters prophage-mediated defense system and the observed recombination is yet unknown. The proposal of Kauffman et al. (2022) that prophage-encoded antiphage defenses may digest genomes of invading phages, yielding fragments that recombine with the prophage, may be applicable in generating BIBs in this system.

### Targeted phage–phage recombination as a strategy to overcome dearth of therapeutic phages

**Fig. 5.**
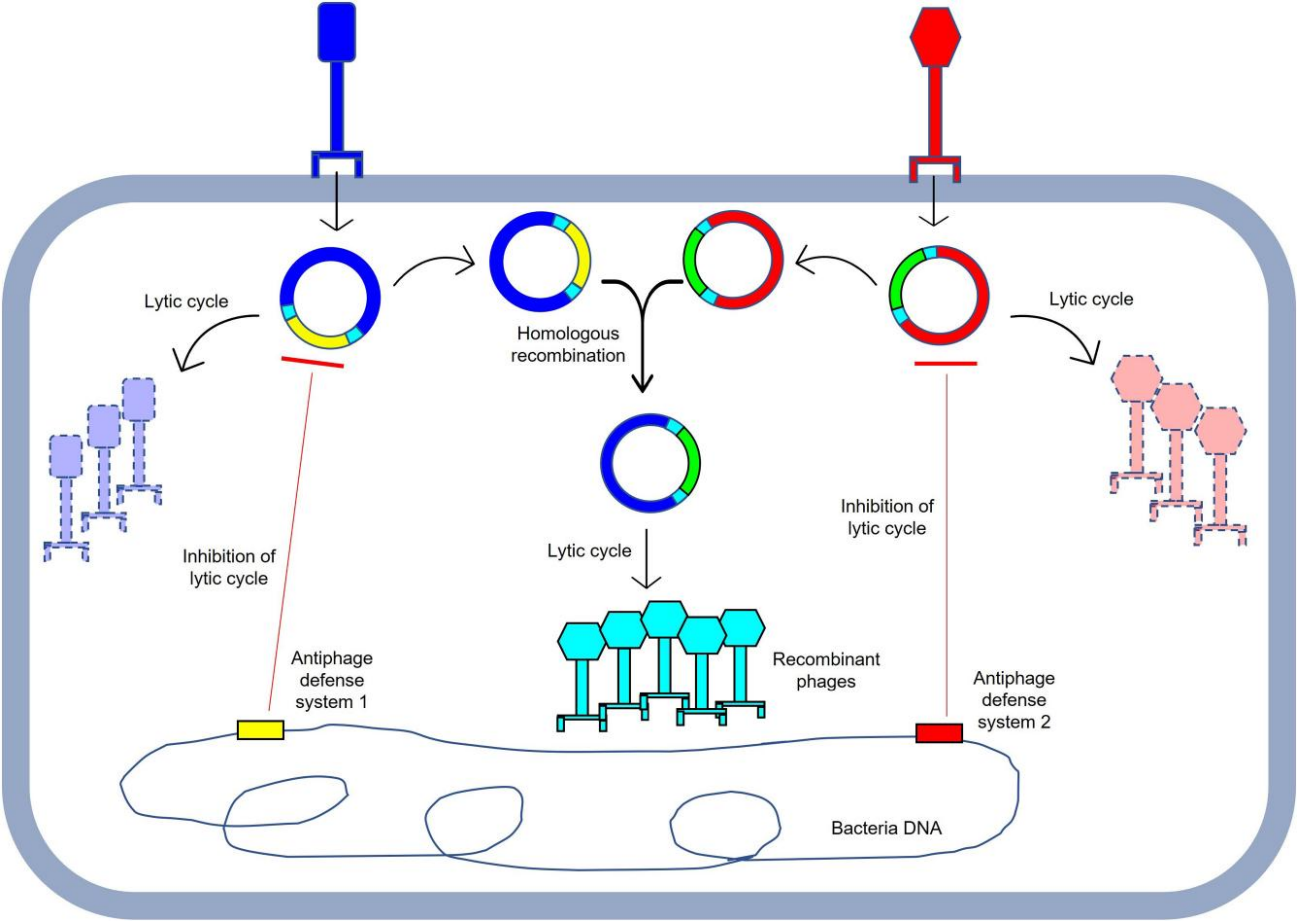
Model showing coinfection as a strategy to overcome antiphage systems. Bacteria (or lysogen) have antiphage defense systems that block lytic infection by both rectangular shaped head (left)- and hexagonal shaped head (right) phages. Antiphage defense system 1 (rectangular block) targets portions of rectangular shaped head phage and antiphage system 2 (rectangular block) targets portions of hexagonal shaped head phage. During coinfection, homologous regions (shown as small blocks flanking antiphage defense systems) may recombine, yielding recombinant phage that escape defense systems present in the cell.

The growing incidence of antibiotic resistance in bacterial pathogens has revived interest in phage therapy. Several cases of emergency use authorization of phages for treatment of drug-resistant infections have been reported with varying degrees of success (Kutateladze and Adamia 2008; Aslam et al. 2019; Dedrick et al. 2019; Law et al. 2019). While promising, it is challenging to obtain phages capable of infecting a given drug-resistant pathogenic bacterial strain (Dedrick et al. 2021, 2023). Typically, determining which phages infect a given bacterial strain is accomplished by plating pure phage lysates on the host. Some bacteria may carry antiphage defense systems capable of blocking any two phages A and B; yet coinfection by phages A and B could result in recombination to yield phages that escape antiphage defense systems (Fig. 5). This strategy could expand the pool of therapeutic phages especially for pathogenic bacteria for which phages are scarce. For pathogenic bacteria harboring resident prophage(s), therapeutic phages might be engineered to elicit recombination with the resident prophage. Our proposed strategy could employ both lytic and temperate phages. Resulting temperate phages that infect a given bacterial candidate for phage therapy could then be engineered to yield lytic phages.

This work provides the foundation for addressing several interesting questions about recombination within mycobacteriophages. (1) Does recombination occur before Butters prophage induction or is the prophage induced before recombination? (2) Are there any Butters prophage- or Island3-specific factors that trigger the observed homologous recombination? (3) What is the source of recombineering enzymes that catalyze the observed recombination? Potential recombineering candidates include the host mc^2^155 RecA, Butters *RecET* system or Island3 *RecET* system (Martinsohn et al. 2008; De Paepe et al. 2014). (4) Can recombination be reproduced within other mycobacteriophage clusters and subclusters? Overall, discovery of these naturally occurring mycobacteriophages that recombine to yield mosaic recombinant phages will allow interrogation of molecular mechanisms underpinning this phenomenon.

## Supplementary Material

jkad210_Supplementary_Data

## Data Availability

GenBank accession numbers for phage genomes in this report are as follows: Butters (KC576783), Island3 (HM152765), Eponine (MN945904), BIB1 (OP961731), BIB2 (OP961730), BIB3 (OP961729), BIB4 (OP961728; Sin et al. 2013), BIB6 (OP961727; Alcoser et al. 2010), BIB7 (OP961726), BIB8 (OP961725; Mansi et al. 2020; Mohammed et al. 2022), BIB9 (OP961724; Mohammed et al. 2022), and BIB10 (OP961723; Mohammed et al. 2022). The sequencing reads for recombinant phages underlying this article are available in BioProjects with accession PRJNA488469 (Mohammed and Ware 2022; Mohammed et al. 2022) for BIBs 1–4 [SRAs: BIB1 (SRX8648491); BIB2 (SRX8648492); BIB3 (SRX8648493); BIB4 (SRX8648494)] and Mohammed HT, Ware VC, Novogene Corporation. 2022. Bioproject: Recombinant Bacteriophage Sequencing Project. Accession: PRJNA906721, ID: 906721. WGS of recombinant phages formed by plating Mycobacteriophage Island3 on Butters lysogen. Sequencing Read Archives: BIB6 (SRX18439511); BIB7 (SRX18439512); BIB8 (SRX18439513); BIB9 (SRX18439514; Russell 2020); BIB10 (SRX18439515; Mohammed and Ware 2022). Supplemental material available at G3 online.
