# Supplementary material for: Characterization of novel recombinant mycobacteriophages derived from homologous recombination between two temperate phages
Source: G3 (Bethesda). 2023 Sep 15;13(12):jkad210. doi: 10.1093/g3journal/jkad210 (PMC10700106; doi:10.1093/g3journal/jkad210)
Supplement: jkad210_Supplementary_Data [file jkad210_supplementary_data.zip › Figure_S1_G3-2023-404481.pdf]

Figure S1A

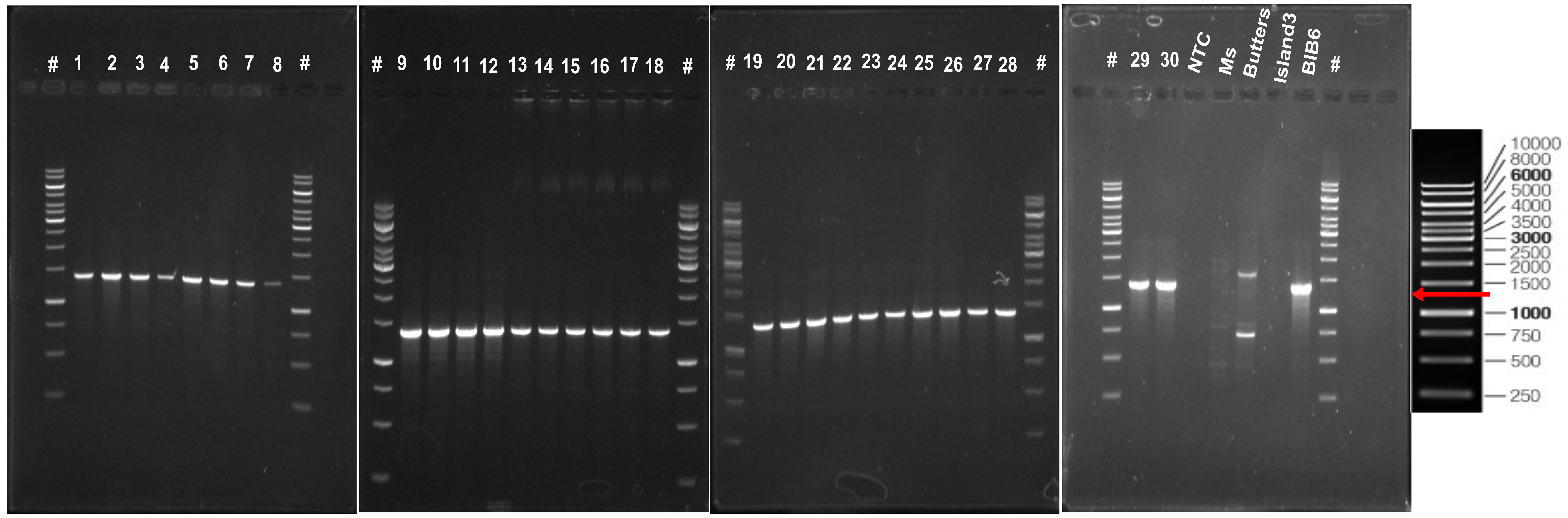

Screening of secondary plaques using BIB-specific primers

Figure S1B

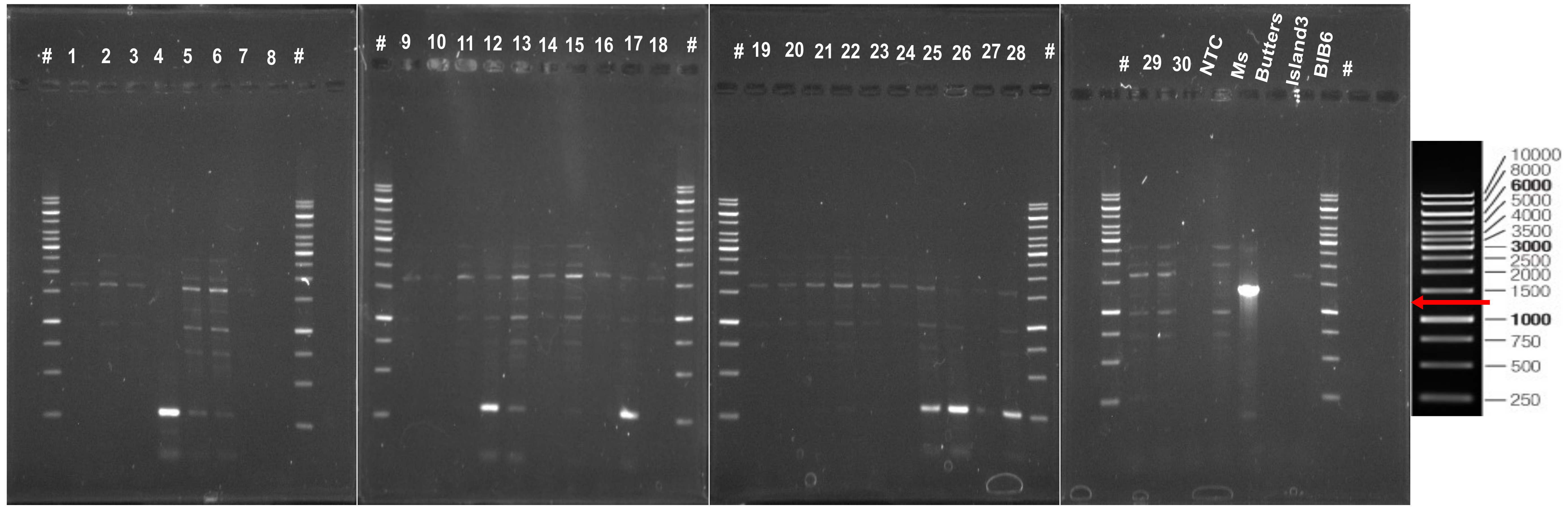

Screening of secondary plaques using Butters-specific primers

Figure S1C

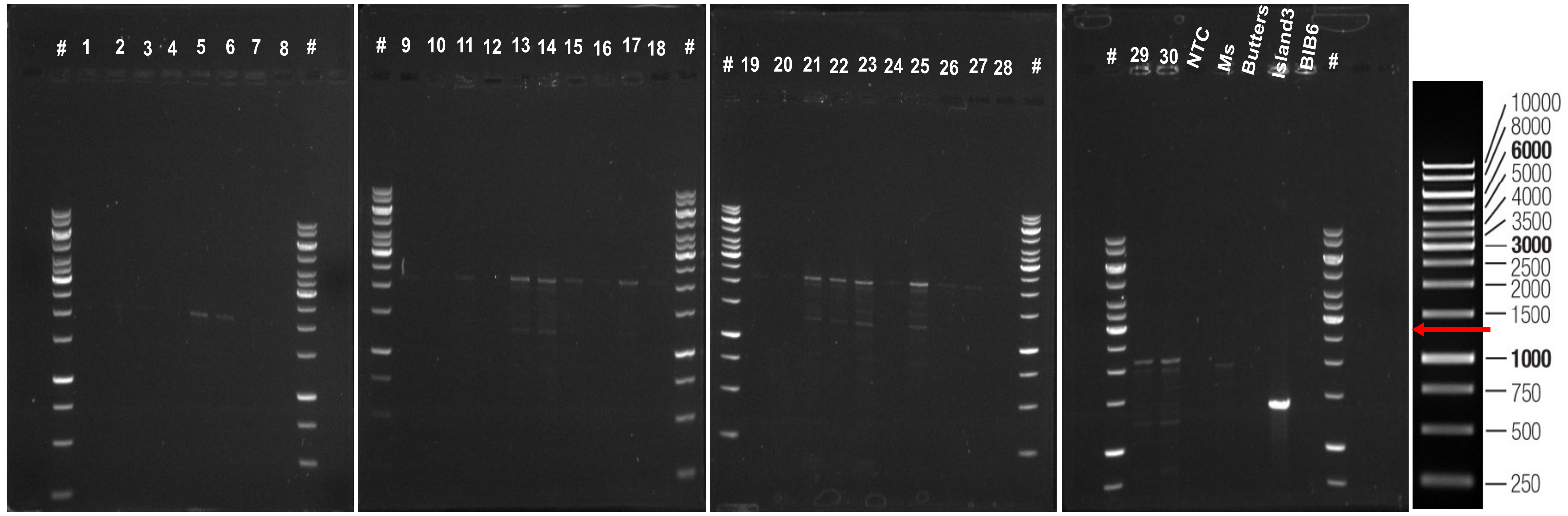

Screening of secondary plaques using Island3-specific primers

Figure S1D

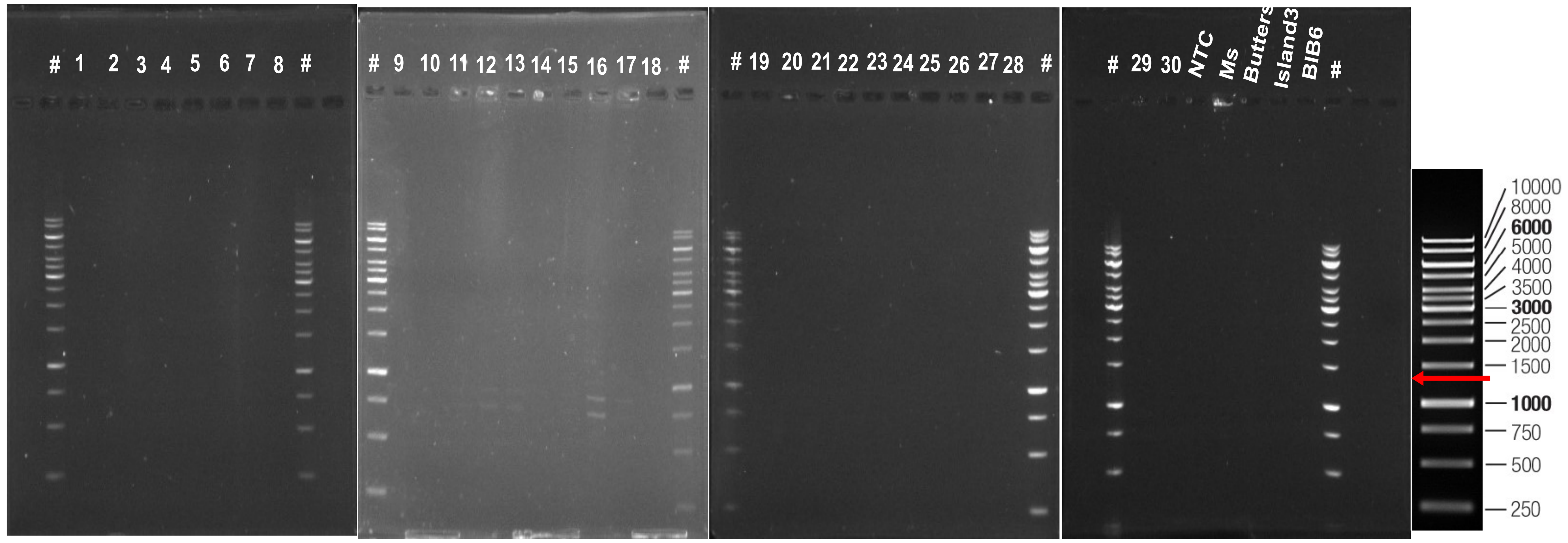

Screening of secondary plaques using IBI-specific primers

Figure S1. PCR screening of secondary plaques obtained by replating primary plaques (related to Figure 4). Each seconday plaque was picked into 100 µL of phage buffer and 1 µL was used as template for PCR. The first three panels contain plaques 1-28. The fourth panel contains plaques 19, 30 no template control (NTC) *Mycobacterium smegmatis* mc2155 (MS), Butters lysate, Island3 lysate and BIB 6 lysate. PCRs were run on 0.8% agarose gel at 60V. GeneRuler 1kb DNA ladder, Thermofisher scientific, was used as reference ladder. Expected product size is 1,367 bp for BIB (red arrow). Non-specific bands appear in some instances due to non-specific primer annealing for PCR conditions used. **A.** PCR performed ujsing BIB-specific primers **B.** PCR performed ujsing Butters-specific primers **C.** PCR performed ujsing Island3-specific primers **D.** PCR performed ujsing IBI-specific primers. Note that all 30 secondary plaques show the expected PCR product with BIB-specific primers but not with Butters- Island3- or IBI-specific primers. This is representative of at least 3 independent biological replicates.
