# Supplementary material for: Characterization of novel recombinant mycobacteriophages derived from homologous recombination between two temperate phages
Source: G3 (Bethesda). 2023 Sep 15;13(12):jkad210. doi: 10.1093/g3journal/jkad210 (PMC10700106; doi:10.1093/g3journal/jkad210)
Supplement: jkad210_Supplementary_Data [file jkad210_supplementary_data.zip › Table_S1_G3-2023-404481.docx]

**Table S1:** List of primers used for Polymerase Chain Reactions (PCRs)

| **Name** | **Sequence (5’ → 3’)** | **Notes** |
| --- | --- | --- |
| F_rr1_Butters_diff | ACCGGCGATCGCGTGCTC | Screening at first homology region. Specific to Butters genome and within gp26. |
| R_rr1_Butters_diff | TCCGGATGGGCGGGCTGG | Screening at first homology region. Specific to Butters genome and within gp27. |
| F_rr1_Island3_diff | ACCGGCGAGCGCGTCAAA | Screening at first homology region. Specific to Island3 genome and within gp29. |
| R_rr1_Island3_diff | ACCGGTCAGCTGCCCGCC | Screening at first homology region. Specific to Island3 genome and within gp30 |
| F_BIB_Island3_46 | ATGAACTGAAACCGCGCAAG | Screening at second homology region. Specific to Island3 genome and within gp46 |
| R_BIB_Butters_54 | CAACTCGCCGATACTGGAGG | Screening at second homology region. Specific to Butters genome and within gp54 |
| F_IBI_Butters_48 | GTTGCGCTCGAACTGTCATC | Screening at second homology region. Specific to Butters genome and within gp48 |
| R_IBI_Island3_51 | TCATCGTCAGCTGTATCCGC | Screening at second homology region. Specific to Island3 genome and within gp51 |
